# Evaluation of Referrals to the Enhanced Team East, South West Yorkshire Partnership Teaching NHS Foundation Trust

**DOI:** 10.1192/bjo.2026.11529

**Published:** 2026-06-30

**Authors:** Julius Akhetuamhen

**Affiliations:** South West Yorkshire Partnership Teaching NHS Foundation Trust, Wakefield, United Kingdom

## Abstract

**Aims:**

To evaluate the appropriateness, quality, and outcomes of referrals to the Enhanced Team East (ETE) in order to ensure that the service is effectively targeted to individuals with complex mental health needs who require intensive, multidisciplinary community support.

**Methods:**

Retrospective review of referrals processed by ETE over the defined period (January 2025 - June 2025), focusing on Single Point of Access (SPA)-received referrals, duty system triage, and MDT decision-making. it was designed to evaluate referral patterns, adherence to inclusion criteria, and outcomes, while identifying opportunities for process improvement.

**Results:**

- A total of 60 referrals were received over the 6-month period, showing steady demand for the Enhanced Team East (ETE). Most of the referrals were from the wards and SPA.
- Only 31.7% were accepted, suggesting many referrals may not meet the threshold or that SPA/teams may need clearer guidance on referral criteria.
- Significant proportions were transferred to Core teams(traditional CMHT) (23.3%) or discharged (18.3%), indicating opportunities for improved triage or alternative pathways before referral.
- The processing time for accepted referral ranged from 7 days to 94 days, with an average of 32.6 days.
- Most of the referrals were from the wa

**Conclusion:**

- The evaluation shows that the Enhanced Team East receives a steady flow of referrals, but only a subset of these referrals meet the threshold for acceptance.
- Processing-time analysis reveals variability in responsiveness, indicating potential workflow delays within triage and allocation processes.
- Improving clarity around referral criteria, strengthening collaboration with referring teams, and reviewing triage processes could enhance the efficiency and appropriateness of referrals.
- Overall, the findings provide a strong basis for targeted service improvements and support the need for future reaudit to measure progress

